# 

**Published:** 1895-06

**Authors:** 


### CONTENTS—JUNE, 1895.—VOL. X., NO. 12.

ORIGINAL COMMUNICATIONS—

Miasmatic Paralytic Fever, or Pernicious Malarial Fever. D. L. Peeples, M. D., Navasota . . . . . . . . . . . . . . . . . . . . . . 629

Report of a Few Surgical Cases, with Remarks on Practical Points. J. B. S. Holmes, M. D., Atlanta, Ga . . . . . . . . . . . . . . . . 637

"Increase of Mental Unsoundness"—A Review of Dr. Wallace's Paper. A. N. Denton, M. D., Austin.. . . . . . . . . . . . . . . 646

Croupous Rhinits. R. H. A. Boyd, A. M., M. D., San Antonio . . . 657

DEPARTMENT OF OPHTHALMOLOGY—

Recent Progress in Ophthalmology. Frank C. Todd, M. D., Fort Worth.. . . . . . . . . . . . . . . . . . . . . . . . . . . . 658

CORRESPONDENCE—

The Mexican Dysentery Remedy Again. Dr. Knox . . . . . . . . . 663

SOCIETY NOTES—

Richmond Academy of Medicine Meeting . . . . . . . . . . . . . 665

EDITORIAL—

The Journal—Close of Volume X . . . . . . . . . . . . . . . . . 667

Do the People Need Protection from Quacks? . . . . . . . . . . . 668

MEDICAL NEWS AND MISCELLANY—

Numerous Paragraphs of Interest . . . . . . . . . . . . . . . . 670

PUBLISHERS' NOTES . . . . . . . . . . . . . . . . . . . . . . . 676

